# The Effectiveness of Two Methods of Prescribing Load on Maximal Strength Development: A Systematic Review

**DOI:** 10.1007/s40279-019-01241-3

**Published:** 2019-12-11

**Authors:** Steve W. Thompson, David Rogerson, Alan Ruddock, Andrew Barnes

**Affiliations:** Academy for Sport and Physical Activity, Sheffield Hallam University, Sheffield, UK

## Abstract

**Background:**

Optimal prescription of resistance exercise load (kg) is essential for the development of maximal strength. Two methods are commonly used in practice with no clear consensus on the most effective approach for the improvement of maximal strength.

**Objective:**

The primary aim of this review was to compare the effectiveness of percentage 1RM (% 1RM) and repetition maximum targets (RM) as load prescription methods for the development of maximal strength.

**Methods:**

Electronic database searches of MEDLINE, SPORTDiscus, Scopus, and CINAHL Complete were conducted in accordance with PRISMA guidelines. Studies were eligible for inclusion if a direct measure of maximal strength was used, a non-training control group was a comparator, the training intervention was > 4 weeks in duration and was replicable, and participants were defined as healthy and between the ages of 18–40. Methodological quality of the studies was evaluated using a modified Downs and Black checklist. Percentage change (%) and 95% confidence intervals (CI) for all strength-based training groups were calculated. Statistical significance (*p* < 0.05) was reported from each study.

**Results:**

Twenty-two studies comprising a total of 761 participants (585 males and 176 females) were found to meet the inclusion criteria. 12 studies were returned for % 1RM, with 10 for RM. All studies showed statistically significant improvements in maximal strength in the training groups (31.3 ± 21.9%; 95% CI 33.1–29.5%). The mean quality rating for all studies was 17.7 ± 2.3. Four studies achieved a good methodological rating, with the remainder classified as moderate.

**Conclusions:**

Both % 1RM and RM are effective tools for improving maximal strength. % 1RM appears to be a better prescriptive method than RM potentially due to a more sophisticated management of residual fatigue. However, large heterogeneity was present within this data. Lower body and multi-joint exercises appear to be more appropriate for developing maximal strength. Greater consensus is required in defining optimal training prescriptions, physiological adaptations, and training status.

**Electronic supplementary material:**

The online version of this article (10.1007/s40279-019-01241-3) contains supplementary material, which is available to authorized users.

## Key Points

**Table:** 

Prescribing load via percentages of 1RM appears to be a better method for improving maximal strength than repetition maximum targets due to a more comprehensive management of residual fatigue.
Multi-joint, compound, lower body exercises elicited a greater improvement in maximal strength than single-joint, isolated, upper body exercises.
Large heterogeneity in training prescriptions, training status, and physiological assessment methods were evident in the literature, with a clear need for greater consensus on the most effective way to improve maximal strength in various demographics.

## Introduction

Resistance training is important for athletic development and is underpinned by 50 + years of peer-reviewed evidence [1–3]. Resistance training is employed to develop maximal strength, but can also be used to enhance speed, agility, rate of force development (RFD), hypertrophy, muscular endurance, motor control, balance, and coordination [1]. Sport-specific technical skills such as jumping, sprinting, and change of direction can also be improved following this type of training [2, 4]. Maximal strength can be defined as one’s ability to exert maximal force against an external resistance and requires a maximal voluntary contraction [3, 5], and is associated with many of the aforementioned physical qualities [4]. Optimising the prescription of resistance training is, therefore, an important consideration for the strength and conditioning practitioner.

Effective resistance training prescription manipulates variables such as training volume and frequency, exercise selection and order, movement velocity, rest periods, and training load [6, 7]. Manipulating these variables elicits specific physiological adaptations such as an increase in neural recruitment or the development of muscle cross-sectional area [8, 9]. These physiological adaptations have been linked with prescription methods used to elicit improvements in maximal strength, specifically the manipulation of training volume and load [8–10].

Optimising load prescription is essential for the effective development of maximal strength [9, 11, 12]. Load can be prescribed using a two-part method: 1) undertaking a dynamic maximal strength test [1 repetition maximum (1RM), for example] and 2) prescribing submaximal percentage loads based upon the initial 1RM (e.g., 85% of 1RM) (% 1RM) or repetition maximum targets (e.g., 5RM) (RM) [6, 7]. Both these methods of load prescription are common in practice and research; however, the most effective in developing maximal strength is still yet to bet determined.

Training programmes based on the % 1RM load prescription method use submaximal percentages based off the maximal load an individual can lift (1RM) [13, 14]. Proponents of this method suggest that it is more favourable than using RM targets when implementing an undulated approach to training due to the ability to prescribe light and heavy days across a week, control for different proximities to failure, and provide a more objective programming strategy for individuals [15, 16]. Conversely, providing individuals with RM targets allows for a more auto-regulatory approach in which the RM target dictates the load [14]. Supporters of this method suggest that due to daily fluctuations in strength based upon a number of factors such as sleep, residual fatigue, and nutritional status; RM targets can provide a more flexible programming strategy than % 1RM and reduce the number of required direct or indirect strength assessments [14]. However, using RM targets, similar to that of more novel methods such as repetitions in reserve (RIR)—the quantification of training intensity by assigning the number of repetitions still able to perform immediately following a working set in accordance with a 1–10 scale of effort (e.g., 1 = 1 rep, 0 = 0 reps etc.)—require the individual to subjectively adjust loads, potentially resulting in inaccurate or inappropriate prescriptions [17, 18].

Comparative charts and tables have previously been designed to provide an interchangeable approach between % 1RM and RM targets [6]. However, research has highlighted that the repetition–load continuum can vary dependent on the population (trained vs. untrained or strength vs. endurance, for example) [19–22]. Descorges et al. [20] highlighted differences in the number of repetitions performed when comparing four different types of athletes (handball vs. powerlifters vs. swimmers vs. rowers). The more strength-based athletes performed significantly lower repetitions across multiple percentages of 1RM compared to the endurance-based athletes. These results were similar to Richens et al. [21]. Repetition maximum targets and repetitions to failure have also been previously provided to predict 1RM [20, 23, 24]. Mayhew et al. [23] investigated 14 different predictive equations and observed differences of − 24.0% to 27.1% in some equations when compared to the direct assessment in bench press. Similarly, Garcia-Ramos et al. [24] compared two predictive equations when lifting to failure in the prone bench-pull, with the largest differences being − 3.6 ± 5.38 kg. The various RM targets associated with different % 1RM values demonstrates that pre-defined rep-load continuums may not be appropriate and the two methods of prescribing training load are not interchangeable with one another, and therefore, their individual effectiveness needs to be assessed.

To date, only one study has directly compared the two aforementioned methods of load prescription [25]. 15 healthy male participants were split into two training groups (relative intensity vs. RM targets) and were asked to complete a volume-equated and exercise-matched 10 week block-periodised resistance training intervention (3 × days per week). The RM group worked to a maximum in each training session (the final set performed must be a true RM), whereas the relative intensity group worked to percentages of the maximum set/repetition combinations. This relative intensity method allowed for the perturbations in strength levels to be accounted for whilst still working to individual percentages of 1RM. Carroll et al. [25] observed greater improvements in vertical jump performance, RFD, and maximal strength (peak force) during an isometric mid-thigh pull assessment in the relative intensity group compared to the RM group. These differences were attributed to a greater training stress in the RM group due to the consistent training to failure prescribed each week. Despite encouraging results in the favour of % 1RM prescriptions, more investigation is required to determine the efficacy of each method, and provide more robust recommendations as to which is the best method to adopt in practice.

The purpose of this review is to assist practitioners’ understanding of methods used to prescribe load. There are a number of prescriptive approaches available to strength and conditioning (S&C) coaches, but to our knowledge, no study has assessed the most effective tool for developing maximal strength. Therefore, the aim of this systematic review was to compare the effectiveness of % 1RM vs. RM prescriptions as a means of improving maximal strength development. A secondary aim of the review was to investigate the quality of research in this area, to develop recommendations for S&C practitioners and researchers in terms of methodological approaches and research designs.

## Methods

This review has been written in accordance with the PRISMA (Preferred Reporting Items for Systematic Reviews and Meta-analyse) statement [26].

### Literature Search

Literature searches were originally performed on 11th October 2017 and then updated on 30th August 2018, 14th March 2019, and 13th September 2019 using the electronic search engines SPORTDiscus, MEDLINE, Scopus, and CINAHL Complete. Searches were performed using titles, abstracts, and keywords, utilising Medical Subject Headings (MeSH), indexing terms, and Boolean operators (AND/OR/NOT). Terms were grouped into themes relating to resistance training, prescriptive methods, and age. For SPORTDiscus, the following search terms were used; ‘resistance exer* or resistance train* or resistance strength* or resistance load*’, ‘musc* strength or strength train*’, ‘Musc* power or power train*’, ‘rate of force development or RFD’, ‘weight lift* or weight train*’, ‘olympic lift*’ AND ‘1RM or rep* max*’, ‘rep* to fatigue or RTF or predict* equation or AMRAP’ NOT ‘senior or elder* or old’, ‘supplement*’, ‘obes* or overweight or blood flow restrict*’, ‘Injur*’. All searches were conducted by the lead author (ST) and developed in consultation with an information scientist. The search strategy was piloted and refined prior to being implemented.

Search results were collated using EndNote software (Thomson Reuters, New York) with duplicates removed automatically (EndNote) and manually (ST). The remaining titles and abstracts were screened for relevance by the lead author. Of those that were deemed potentially relevant, full texts were obtained and independently assessed for eligibility by the lead author, with a random sample (10%) independently assessed by two of the research team (DR, AB). The included studies were then independently assessed by a second author (AR). If the inclusion of a study could not be agreed upon, a third author facilitated a discussion to reach a consensus. Reference lists of each study were manually searched to identify potentially relevant studies (ST).

### Inclusion and Exclusion Criteria

Studies were deemed eligible if they met the following criteria:

- A direct, practical measure of strength was employed (1RM).
- A non-resistance training, control group was used as a comparator.
- The control group continued normal daily activities without additional exercise that would influence strength.
- The training intervention was progressive.
- The methods section contained sufficient information for the training intervention to be fully replicable.
- The training intervention was strength-based, isotonic exercise lasting for a minimum of 4 weeks.
- No form of concurrent training was prescribed (plyometric and/or endurance).
- Participants were aged 18–40.
- Full texts were available in English and were original, peer-reviewed, and primary research.

Studies were not excluded based on the sex of the participants or previous training history. This review did not control for volume matching. It was thought with the focus being prescribing load, only including studies that also matched training volumes would reduce the inclusivity of the search. In the event a study used multiple groups and only some conditions met the inclusion criteria, only the relevant data was extracted.

### Data Extraction Process

Study characteristics including sample size (*n*), age (years), body mass (kg), stature (cm), sex, training history, duration of the intervention, training frequency, description of the intervention (exercises, sets, reps, rest, and load), direct assessment of strength, and method of programme progression were extracted from the eligible studies. The means and standard deviations (SD) for the primary outcome measure [change in absolute strength (kg)] were obtained and relative changes [percentage difference (% diff)] calculated with 95% confidence intervals. All strength data were reported in absolute values (kg) unless unavailable, in which case relative (1RM/body mass) values were reported. Data extraction of all articles was independently assessed for accuracy (AR). When relevant data were not reported, authors were contacted. If authors failed to provide the necessary data, pixel analysis was used to extract appropriate values (DigitizeIt) (ST). Reviewers were not blinded at any stage of the validation process.

### Methodological Quality Assessment

Methodological quality was assessed using the Downs and Black [27] quality assessment tool, as modified by Davies et al. [28]. This quality assessment tool was deemed more appropriate than other tools (Cochrane and PEDro, for example) due to its greater suitability to a non-clinical intervention [29–32]. A detailed description of each criterion can be found elsewhere [27]. Briefly, of the 29 points available, 20 + was deemed as a ‘good’ methodology, 11–19 moderate, and < 11 as poor quality. This process was independently assessed by two authors (ST/AR). Any disputes were settled through discussion with a third author (DR).

## Results

### Description of Studies

**Fig. 1:**
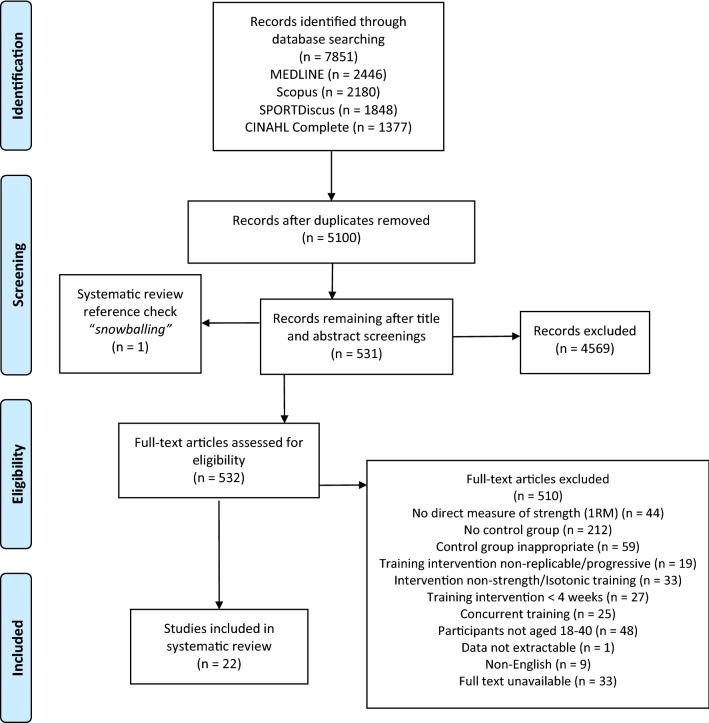
PRISMA flow diagram. *Snowballing* studies included for eligibility assessment from other, relevant systematic reviews

Figure 1 details the PRISMA [30] flow chart. A total of 22 studies [33–54], totalling 761 participants (585 males and 176 females), were eligible for review. Sample sizes ranged from 17 to 120 participants, with numbers for experimental and control groups ranging from 5 to 47 participants. Mean ages ranged from 20.0 ± 1.8 to 31.6 ± 9.8 across all studies (see Table 1 for all participant characteristics). Of the two prescriptive methods (% 1RM, and RM), 12 studies utilised the % 1RM prescriptive approach and 10 employed the RM prescriptive approach.

**Table 1:** Study characteristics

Study	Participants (*n*)	Groups (+ participant numbers (*n*))	Sex (*n*)	Age (years ± SD)	Body mass (kg ± SD)	Stature (cm ± SD)	Resistance training experience	Participant characterisation
Weiss et al. (1988) [33]	56	RT (28)	M (28)	20.8 ± 1.8	NR	NR	NRT < 3 months	Healthy
		C (28)	F (28)					
Braith et al. (1993) [34]	58	RT (47)	M (33)	24.0 ± 4.0	70.1 ± 9.0	174.0 ± 6.3	NRT < 1 year	Untrained
		C (11)	F (25)	25.0 ± 5.0	74.3 ± 14.5	172.6 ± 6.6		
Moss et al. (1997) [35]	31	RT—G90 (9)	M	22.7 ± 3.4	75.8 ± 5.6	179.0 ± 6.8	Well-trained	University students (non-dominant arm = control group)
		RT—G35 (11)		24.0 ± 3.4	83.2 ± 8.8	185.7 ± 8.5		
		RT—G15 (10)		22.9 ± 2.8	78.1 ± 10.4	182.6 ± 6.7		
Bell et al. (2000) [36]	21	RT (11)	M (12)	22.3 ± 3.3	73.4 ± 11.6	176.0 ± 9.3	NRT	University students
		C (10)	F (9)					
Campos et al. (2002) [37]	31	RT—LR (9)	M	21.1 ± 1.5	80.1 ± 8.4	179.8 ± 6.5	NRT < 6 months	Healthy
		RT—IR (7)		20.7 ± 2.9	79.5 ± 7.8	179.6 ± 7.4		
		RT—HR (7)		20.4 ± 3.5	70.2 ± 9.5	174.3 ± 8.6		
		C (5)		31.6 ± 9.8	80.8 ± 23.3	178.1 ± 5.5		
McBride et al. (2003) [38]	28	RT—S1 (9)	F (13) M (15)	22.1 ± 3.4	83.7 ± 29.4	172.8 ± 10.5	NRT (< 6 months)	Untrained
		RT—M6 (9)		20.0 ± 1.22	70.7 ± 23.0	169.4 ± 11.8		
		C (10)		22.4 ± 1.89	70.6 ± 7.8	171.3 ± 7.2		
Willoughby (2004) [39]	22	RT (12)	M	20.9 ± 2.76	78.7 ± 6.2	176.5 ± 7.1	NRT < 6 months	Untrained
		C (10)						
Tricoli et al. (2005) [40]	14	RT (7)	M	22.0 ± 1.5	73.4 ± 10.4	179.4 ± 8.8	NRT < 3 months (trained prior)	College students
		C (7)						
Rana et al. (2008) [41]	16	RT (9)	F	20.6 ± 1.9	64.1 ± 7.9	165.6 ± 4.9	NRT	Untrained
		C (7)		22.9 ± 2.4	72.5 ± 15.0	163.6 ± 4.5		
Tanimoto et al. (2008) [42]	24	RT (12)	M	19.5 ± 0.5	63.8 ± 4.0	174.8 ± 4.3	NRT	Healthy
		C (12)		19.8 ± 0.7	64.2 ± 4.0	174.3 ± 7.2		
Terzis et al. (2008) [43]	17	RT (11)	M	22.0 ± 1.0	85.0 ± 4.0	184.0 ± 3.0	NRT < 1 year	P.E students
		C (6)						
Hartmann et al. (2009) [44]	40	RT—SPP (13)	M	24.31 ± 3.2	84.7 ± 11.2	183.9 ± 7.2	RT in BP (minimum 1RM of 100 kg)	Sport science students
		RT—UP (14)		25.14 ± 4.0	79.4 ± 10.4	177.6 ± 7.5		
		C (13)		24.77 ± 3.1	74.4 ± 12.1	180.5 ± 8.1		
Cormie et al. (2010) [45]	16	RT (8)	M	23.9 ± 4.8	79.8 ± 12.0	180.0 ± 6.4	NRT (Technically proficient in BS)	Healthy
		C (8)						
Chtourou et al. (2012) [46]	30	RT—MTG (10)	M	22.9 ± 1.3	72.0 ± 8.8	180.0 ± 5.0	NRT < 6 months	P.E students
		RT—ETG (10)						
		C (10)						
Weier et al. (2012) [47]	12	RT (6)	M (6) F (6)	20 ± 0.8	NR	NR	NR	University students
		C (6)		22 ± 0.6				
Naclerio et al. (2013) [48]	32	RT—LV (6)	M (20) F (12)	23.3 ± 1.2	66.4 ± 11.0	169.9 ± 8.4	NRT < 5 years	Team sports athletes Soccer (20) (M) Volleyball (12) (F)
		RT—MV (6)		23.3 ± 1.4	71.4 ± 8.5	173.3 ± 7.6		
		RT—HV (8)		23.9 ± 2.0	69.4 ± 12.5	173.0 ± 9.8		
		C (7)		22.1 ± 1.1	71.1 ± 14.2	169.7 ± 6.9		
Aguiar et al. (2015) [49]	18	RT (9)	M	20.9 ± 2.0	73.7 ± 9.4	173.8 ± 6.9	NRT < 6 months	Healthy
		C (9)		20.0 ± 1.8	75.0 ± 8.8	176.4 ± 8.1		
Akagi et al. (2016) [50]	23	RT (13) C (10)	M	22.1 ± 1.1	61.4 ± 5.8	170.6 ± 5.8	NRT upper body (< 6 months)	Healthy
Botton et al. (2016) [51]	43	RT—UG (14)	F	24.8 ± 1.4	60.8 ± 6.4	163.0 ± 6.5	NRT < 3 months	Healthy
		RT—BG (15)		24.3 ± 3.7	57.0 ± 4.8	160.2 ± 5.8		
		C (14)		22.7 ± 2.8	58.0 ± 5.7	163.6 ± 6.2		
Wirth et al. (2016) [52]	120	RT—SQ (43)	M	23.7 ± 2.7	81.6 ± 9.8	181.7 ± 7.5	NR	Students
		RT—LP (40)		23.8 ± 2.3	80.5 ± 8.1	180.1 ± 7.0		
		C (37)		25.1 ± 2.1	78.2 ± 8.5	181.0 ± 5.7		
Jarvis et al. (2017) [53]	21	RT (11) C (10)	M (15) F (6)	27.5 ± 3.2	72.7 ± 18.0	169.6 ± 10.3	RT > 1 year	Collegiate athletes
				27.2 ± 3.4	76.4 ± 11.5	176.2 ± 7.9		
Souza et al. (2018) [54]	33	RT—NP (8)	M	25.6 ± 6.3	79.5 ± 13.0	172.8 ± 6.1	NRT (< 6 months)	College students
		RT—TP (9)		25.0 ± 7.0	76.0 ± 9.9	175.3 ± 5.7		
		RT—UP (8)		24.4 ± 5.2	74.9 ± 4.2	176.8 ± 5.3		
		C (8)		25.1 ± 3.3	76.8 ± 11.7	173.6 ± 6.8		

*Mean ± SD* standard deviation

*1RM* 1 repetition maximum, *BG* bilateral training group, *C* control, *cm* centimetres, *BP* bench press, *BS* back squat, *ETG* evening training group, *F* female, *G15* 15% load group, *G35* 35% load group, *G90* 90% load group, *HR* high-repetition group, *HV* high volume, *IR* intermediate-repetition group, *kg* kilograms, *LP* leg press group, *LR* low-repetition group, *LV* low volume, *M* male, *M6* six set training group, *MTG* morning training group, *MV* moderate volume, *n* number, *NP* non-periodised group, *NRT* no resistance training, *NR* not reported, *P.E* Physical Education, *RT* resistance training, *S1* 1 set training group, *SPP* strength-power periodisation, *SQ* squat group, *TP* traditional periodisation group, *UG* unilateral training group, *UP* daily undulating periodised group

15 studies assessed lower body strength (seated plantar-flexion, knee extension, knee flexion, leg press, back squat, half squat, Clean and Jerk, and hip thrust) three studies assessed upper body strength (bicep curl, triceps extension and bench press) [35, 44, 50] and the remaining four assessed a combination of upper and lower body strength (bicep curl, leg press, back squat, latissimus-dorsi pull-down, ab-board, back extension, and upright row) [38, 42, 43, 48]. There was an 11.9% greater improvement in maximal strength when assessing upper body vs. lower body exercises (Table 4). All studies reported pre-and post-intervention data for experimental and control groups (see Table 2 for all training intervention details).

**Table 2:** Training parameters

Study	Duration (weeks)	Frequency (session × week)	Exercise(s)	Sets (*n*)	Reps (*n*)	Rest (min)	Load (% 1 RM or RM target)	Load adjustment
Weiss et al. (1988) [33]	8	3	Seated plantar flexion	4	9–13	2–3	9–13 RM	> When RM target exceeded
Braith et al. (1993) [34]	18	2–3	Knee extension	1	7–10	NR	7–10 RM	> 5% when RM target exceeded
Moss et al. (1997) [35]	9	3	Elbow flexion	3–5	10 (G15) 7 (G35) 2 (G90)	NR	G15 (15%) G35 (35%) G90 (90%)	1RM @ 4 weeks
Bell et al. (2000) [36]	12	3	Bl leg press, UL knee flexion, UL knee extension, bl calf raises	2–6	4–12	NR	72–84%	> Approx. 4% every 3 weeks
Campos et al. (2002) [37]	8	2 × week 1–4 3 × week 5–8	Leg press, back squat, knee extension	LR (4) IR (3) HR (2)	3–5 9–11 20–28	3 2 1	3–5 RM 9–11 RM 20–28 RM	> When RM target exceeded
McBride et al. (2003) [38]	12	2	Bicep curl, leg press, chest flye, sit ups, back extension	1–6 1–3	6–10 15	2–3	6–10 RM	> when RM target exceeded
Willoughby (2004) [39]	12	3	Leg press, knee extension, knee flexion	3	6–8	1.5	85–90%	1RM @ Weeks 3, 6, 9, 12
Tricoli et al. (2005) [40]	8	3	High pull, power clean, clean and jerk, half–squat	3–6	4–6	NR	4–6 RM	Volume increased after 4 weeks
Rana et al. (2008) [41]	6	2 × week 1 3 × weeks 2–6	Leg press, knee extension, back squat	3	6–10	2	80–85%	> When RM target exceeded
Tanimoto et al. (2008) [42]	13	2	Back squat, bench press, latissimus-dorsi pull down, abdominal bend, back extension	3 (+ 1 WU set)	8	1	80–90%	1RM @ 7 weeks
Terzis et al. (2008) [43]	14	2 × week 1–2 3 × week 3–14	Leg press (45° inclination), semi-squat (knees 90°), bench press, arm curl, overhead press, elbow extension (pulley), seated row, sit ups, back extension	2–3	6–20	NR	8–10 RM 6 RM	Daily > to meet RM target
Hartmann et al. (2009) [44]	14	3	Bench press	5	3–25	1.5–5	3–5 RM 8–12 RM 20–25 RM	> 2–10 kg when RM target exceeded
Cormie et al. (2010) [45]	10	3	Back squat	3–7	3–6	3–5	75–90%	1RM @ Week 5
Chtourou et al. (2012) [46]	8	3	Knee extension, knee flexion, back squat	3–6	3–6	2–9	60–120%	1RM @ Week 4
Weier et al. (2012) [47]	4	3	Back squat	6–9	6–8	3	80%	> 2–5% when target exceeded
Naclerio et al. (2013) [48]	6	3	Tp 1: Bench press, incline bench press, dumbbell fly, upright row, lateral raise, Posterior lateral raise, barbell bicep curl Dumbbell bicep curl, machine bicep curl Tp 2: Smith machine parallel squat, leg press, knee extension, latissimus dorsi pull down, seated row, sa dumbbell row, machine triceps extension, standing triceps pushdown, sa triceps extension	1–3	8	3	75%	NR
Aguiar et al. (2015) [49]	8	2	Knee extension	3	8–12	1	75%	1RM @ 15 day intervals
Akagi et al. (2016) [50]	6	3	Triceps extension	5	8	1.5	80%	1RM every 2 weeks
Botton et al. (2016) [51]	8	2	Ul knee extension Bl knee extension	2–4	5–15	1–3	12–15 RM 9–12 RM 7–10 RM 5–8 RM	1–5 kg when RM target exceeded
Wirth et al. (2016) [52]	8	2	Back squat, leg press	5	4–10	5	8–10 RM 6–8 RM 4–6 RM	> 2.5–10 kg when RM target exceeded
Jarvis et al. (2017) [53]	8	3	Hip thrust	5	5	3	85%	> 2.5% when RM target exceeded
Souza et al. (2018) [54]	12	2	Back squat Knee extension	2–4	4–12	2–3	4–12 RM	1RM @ weeks 1, 6, 12

*1RM* 1 repetition maximum, BL bilateral, *G15* 15% load group, *G35* 35% load group, *G90* 90% load group, *HR* high–repetition group, *IR* intermediate–repetition group, *kg* kilograms *LR* low-repetition group, *min* minutes, *n* number, *NR* not reported, *RM* repetition maximum, *UL* unilateral, *SA* single arm, *WU* warm up

### Improvements in Maximal Strength

A summary of the strength developments can be found in Table 3. All 22 studies documented statistically significant (*P *< 0.05) improvements in maximal strength for the training groups (31.3 ± 21.9%; 95% CI 33.1–29.5%) in comparison with their respective control groups (3.4 ± 4.3%; 95% CI 3.9–2.9%); 20 studies presented data in absolute values (kg), with two reporting relative (1RM/body mass) (Table 3).

**Table 3:** Summary of the changes in maximal strength following an intervention compared to a non-training control

Study	Groups	Test	Experimental group(s)			Control Group		
			Pre	Post	Percentage change (%)	Pre	Post	Percentage change (%)
			kg ± SD	kg ± SD		kg ± SD	Kg ± SD	
Weiss et al. (1988) [33]	RT (M)	Seated plantar flexion	98.5 ± 16.5	113.5 ± 13.3	15.2	91.9 ± 18.6	91.9 ± 19.8	0.0
	RT (F)		81.0 ± 23.8	93.4 ± 22.8	15.3	74.4 ± 8.1	74.4 ± 8.1	0.0
Braith et al. (1993) [34]	RT	Knee extension	85.4 ± 27.9	111.6 ± 33.6	30.7	97.2 ± 29.7	100.6 ± 32.0	3.5
Moss et al. (1997) [35]	RT (G90)	Elbow flexion	18.8 ± 3.0	21.7 ± 3.3	15.4	19.4 ± 3.1	20.7 ± 2.8	6.9
	RT (G35)		20.0 ± 4.7	22.0 ± 5.1	10.0	21.0 ± 4.0	21.4 ± 4.2	2.1
	RT (G15)		19.0 ± 4.5	20.3 ± 5.0	6.8	19.8 ± 4.8	21.0 ± 4.7	6.0
Bell et al. (2000) [36]	RT	Knee extension	17.3 ± 2.8	27.3 ± 4.6	57.8	18.2 ± 4.0	20.0 ± 4.0	9.9
		Leg press	151.4 ± 51.8	249.1 ± 151.0	64.5	165.9 ± 67.1	180.0 ± 36.7	8.5
		Knee extension	36.8 ± 9.5	48.6 ± 9.5	32.1	38.2 ± 9.2	39.5 ± 8.1	3.4
		Leg press	260.5 ± 78.1	393.6 ± 75.7	51.1	266.8 ± 104.7	297.3 ± 106.7	11.4
Campos et al. (2002) [37]	RT (LR)	Leg press	309.1 ± 65.9	497.2 ± 93.1	60.8	284.8 ± 38.1	302.6 ± 40.7	6.3
	RT (IR)		292.4 ± 44.4	396.7 ± 68.8	35.7			
	RT (HR)		298.6 ± 35.0	361.9 ± 37.5	21.2			
	RT (LR)	Leg extension	96.1 ± 24.2	154.2 ± 33.3	60.4	93.9 ± 22.9	99.6 ± 24.2	6.1
	RT (IR)		97.5 ± 16.0	144.9 ± 28.8	48.6			
	RT (HR)		86.8 ± 19.7	135.6 ± 11.4	56.2			
	RT (LR)	Back squat	115.2 ± 30.0	246.5 ± 57.0	114.0	116.8 ± 18.2	139.3 ± 23.6	19.3
	RT (IR)		120.34 ± 21.9	213.4 ± 27.7	77.3			
	RT (HR)		111.2 ± 22.0	193.1 ± 20.2	73.7			
McBride et al. (2003) [38]	RT (S1)	Bicep curl	33.8 ± 12.6	37.1 ± 15.1	9.7			
		Leg press	242.9 ± 139.6	324.2 ± 166.4	33.5			
	RT (M6)	Bicep curl	29.6 ± 10.3	35.6 ± 10.8	20.5	30.2 ± 11.2	30.2 ± 11.2	0.0
		Leg press	191.2 ± 76.8	293.4 ± 126.2	53.5	198.2 ± 52.1	208.4 ± 61.7	5.2
Willoughby (2004) [39]	RT	Leg press	3.1 ± 4.2	4.5 ± 5.5	41.4	3.3 ± 4.3	3.8 ± 4.8	15.2
Tricoli et al. (2005) [40]	RT	Half squat	146.3 ± 30.5	210.3 ± 22.3	43.8	149.5 ± 24.6	159.1 ± 22.2	6.4
		Clean & jerk	57.4 ± 5.8	77.4 ± 11.7	34.8			
Rana et al. (2008) [41]	RT	Leg press	198.1 ± 27.2	319.0 ± 52.5	61.1	216.0 ± 36.6	228.8 ± 45.8	5.9
		Back squat	56.7 ± 8.7	83.1 ± 17.4	46.7	60.7 ± 9.1	60.1 ± 31.3	− 0.9
		Knee extension	51.2 ± 10.9	77.1 ± 11.8	50.7	59.5 ± 14.3	62.9 ± 18.0	5.7
Tanimoto et al. (2008) [42]	RT	Vertical squat	105.1 ± 16.1	136.5 ± 20.4	29.9	113.7 ± 16.3	112.9 ± 17.8	− 0.7
		Chest press	41.3 ± 5.4	55.1 ± 9.1	33.4	46.1 ± 10.0	47.3 ± 11.1	2.6
		Lat pull-down	39.6 ± 7.2	55.7 ± 9.0	40.7	47.7 ± 6.9	48.9 ± 7.3	2.5
		Ab-board	59.3 ± 8.8	90.4 ± 13.4	52.5	66.4 ± 7.9	67.1 ± 8.5	1.1
		Back extension	61.5 ± 10.0	113.0 ± 13.5	83.7	70.0 ± 16.4	72.4 ± 16.2	3.4
Terzis et al. (2008) [43]	RT	Back squat	101.0 ± 6.0	123.0 ± 6.0	21.8			
		Leg press	237.0 ± 16.0	297.0 ± 18.0	25.3			
		Bench press	77.0 ± 4.0	90.0 ± 5.0	16.9			
Hartmann et al. (2009) [44]	RT (SPP)	Bench press	95.5 ± 20.9	109.4 ± 19.6	14.5	58.5 ± 10.2	59.2 ± 10.5	1.3
	RT (UP)		95.9 ± 17.5	105.4 ± 19.5	9.9			
Cormie et al. (2010) [45]	RT	Back squat	1.3 ± 0.2	1.6 ± 0.1	28.1	1.4 ± 0.1	1.4 ± 0.1	− 1.5
Chtourou et al. (2012) [46]	RT (MTG) (07:00)	Leg extension	71.0 ± 9.9	87.5 ± 7.9	23.2			
		Leg curl	70.0 ± 11.3	85.5 ± 9.0	22.1			
		Back squat	74.0 ± 12.0	89.5 ± 9.8	21.0			
	RT (MTG) (17:00)	Leg extension	73.5 ± 8.5	87.0 ± 8.2	18.4	69.0 ± 9.7	69.5 ± 9.3	0.7
		Leg curl	73.0 ± 11.1	85.0 ± 7.5	16.4	64.0 ± 9.4	64.0 ± 6.6	0.0
		Back squat	76.5 ± 11.1	88.5 ± 8.5	15.7	67.5 ± 10.3	67.0 ± 9.5	− 0.7
	RT (ETG) (07:00)	Leg extension	69.5 ± 8.0	81.5 ± 4.7	17.3			
		Leg curl	68.5 ± 10.0	81.5 ± 6.7	19.0			
		Back squat	68.0 ± 11.1	80.5 ± 9.8	18.4			
	RT (ETG) (17:00)	Leg extension	72.0 ± 7.5	85.0 ± 4.7	18.1	72.0 ± 9.2	72.0 ± 8.9	0.0
		Leg curl	71.0 ± 8.8	85.0 ± 6.7	19.7	66.5 ± 10.6	67.0 ± 10.1	0.8
		Back squat	71.0 ± 10.5	84.5 ± 9.6	19.0	69.0 ± 10.2	69.5 ± 9.8	0.7
Weier et al. (2012) [47]	RT	Back squat	86.3 ± 13.4	161.6 ± 23.2	87.3	83.1 ± 13.8	85.2 ± 13.9	2.5
Naclerio et al. (2013) [48]	RT (LV)	Bench press	49.3 ± 19.1	54.4 ± 22.1	10.3			
		Upright row	40.8 ± 10.7	45.0 ± 13.8	10.3			
		Back squat	103.0 ± 30.8	107.1 ± 30.6	4.0			
	RT (MV)	Bench press	65.9 ± 24.5	72.0 ± 28.4	9.3			
		Upright row	44.2 ± 9.9	49.9 ± 12.9	12.9			
		Back squat	126.3 ± 29.2	129.8 ± 40.6	2.8			
	RT (HV)	Bench press	46.7 ± 19.6	54.5 ± 18.2	16.7	44.6 ± 21.0	44.1 ± 21.9	− 1.1
		Upright row	38.9 ± 10.7	45.7 ± 13.5	17.5	35.4 ± 12.2	35.9 ± 11.7	1.4
		Back squat	102.1 ± 26.7	119.8 ± 33.6	17.3	100.7 ± 45.0	101.3 ± 43.9	0.6
Aguiar et al. (2015) [49]	RT	Knee extension	107.4 ± 3.9	135.8 ± 5.5	26.4	106.4 ± 2.6	106.9 ± 2.8	0.5
Akagi et al. (2016) [50]	RT	Tricep extension	8.6 ± 1.3	11.5 ± 1.8	33.7	9.1 ± 2.0	9.4 ± 2.3	3.3
Botton et al. (2016) [51]	RT (UG)	Bl knee extension	39.0 ± 7.3	46.6 ± 7.2	19.5			
		Ul knee extension	38.0 ± 7.8	50.2 ± 8.3	32.1			
	RT (BG)	Bl knee extension	35.7 ± 7.6	45.5 ± 8.0	27.5	36.7 ± 8.1	37.0 ± 9.6	0.8
		Ul knee extension	34.9 ± 6.8	43.1 ± 7.3	23.5	39.1 ± 10.0	39.2 ± 10.2	0.3
Wirth et al. (2016) [52]	RT (SQ)	Back squat	97.1 ± 29.0	118.0 ± 29.4	21.5	75.6 ± 23.9	75.9 ± 21.0	0.4
	RT (LP)	Leg press	230.3 ± 57.4	296.8 ± 68.3	28.9	220.7 ± 88.1	226.9 ± 64.7	2.8
Jarvis et al. (2017) [53]	RT	Hip thrust	161.8 ± 50.4	205.9 ± 63.3	27.3	164.6 ± 36.7	174.0 ± 41.9	5.7
Souza et al. (2018) [54]	RT (NP)	Back squat	140.8 ± 23.9	171.0 ± 36.9	21.5	126.8 ± 21.3	132.1 ± 20.1	4.1
	RT (TP)		141.2 ± 19.6	166.4 ± 30.3	17.9			
	RT (UP)		149.6 ± 34.7	178.4 ± 36.8	19.2			

Mean ± SD

*BG* bilateral training group, *BL* bilateral, *ETG* evening training group, *F* female, *G15* 15% load group, *G35* 35% load group, *G90* 90% load group, *HR* high-repetition group, *HV* high volume, *IR* intermediate-repetition group, *kg* kilograms, *LP* leg press group, *LR* low-repetition group, *LV* low volume, *M* male, *M6* six set training group, *MTG* morning training group, *MV* moderate volume, *NP* non-periodised group,, *RT* resistance training, *S1* 1 set training group, *SD* standard deviation, *SPP* strength-power periodisation, *SQ* squat group, *TP* traditional periodisation group, *UG* unilateral training group, *UP* daily undulating periodised group

**Table 4:** Sensitivity analysis comparing maximal strength development across four methodological approaches

	Prescriptive method		Exercise type		Exercise focus		Training duration (weeks)		
	% 1RM	*RM	Compound	Isolation	Upper body	Lower body	6	12	18
Sample size (*n*)	313	448	523	450	207	667	101	509	151
Mean increase in strength (%)	28.8	24.2	33.8	28.4	22.4	34.3	27.2	32.1	32.7
SD (%)	20.2	10.8	24.4	18.0	19.3	21.6	25.2	21.2	20.8
CI upper (%)	31.4	25.4	35.9	30.0	25.0	36.0	32.1	34.0	36.0
CI lower (%)	26.2	23.1	31.7	26.7	19.7	32.7	22.3	30.3	29.3

*Data for RM group and subsequent sub-analyses does not include data presented in Campos et al. [37]

*1RM* 1 repetition maximum, *CI* Confidence Intervals, *RM* Repetition Maximum, *SD* standard deviation

The training groups utilising a percentage-based load prescription significantly improved maximal strength by 28.8 ± 20.2% (95% CI 31.4–26.2%) compared to 34.5 ± 23.5% (95% CI 37.0–32.0%) for the training groups utilising a repetition maximum based load prescription (*p *< 0.05) (Table 4). When removing data derived from Campos et al. [37], which were seemingly outliers and skewed the data, maximal strength increased by 24.2 ± 10.81% (95% CI 23.1–15.4%) for the repetition maximum-based load prescriptive method.

### Periodised Approaches

Five studies employed a periodised approach to their programming (daily undulating, linear or block) [35, 36, 40, 45, 46]. Twelve studies adjusted load by an auto-regulatory increase when a target was met (RM or %) [33, 34, 36–38, 41, 43, 44, 47, 51–53]; 8 studies employed mid-point 1RM tests (ranging from every 2–6 weeks) [35, 39, 42, 45, 46, 49, 50, 54]; 1 study did not report how they adjusted load [48]; and 1 study increased the volume, but kept the load constant [40].

### Training Variables

Training interventions ranged from 4 to 18 weeks across all studies, with 2–3 sessions per week being prescribed. Further analysis detailed a 4.9–5.5% greater improvement in maximal strength, measured via direct 1RM assessments in multiple movements/exercises across all 22 studies when prescribing an intervention over a longer duration (> 6 weeks). The magnitude of the improvements, however, decreased after 6 weeks (Table 4). Nine studies implemented an intervention containing only one exercise [33–35, 44, 45, 47, 49, 50, 53], with four of those employing a multi-joint exercise (e.g., back squat) [44, 45, 47, 53]. 11 studies employed between two and five exercises within the intervention [36–42, 46, 51, 52, 54], with two studies prescribing more than five [43, 48]. Six studies employed single joint or isolated exercises only [33–35, 49–51], with the rest prescribing multi-joint or a combination of the two. Maximal strength increased by 5.4% more in multi-joint, compound exercises compared to single-joint, isolation exercises (Table 4). Exercise specifics for the training groups were 1–6 sets of 3–28 reps, with 1–5 min rest periods. Training intensities ranged from 15 to 120% baseline 1RM-testing scores or 3–28RM. All studies either employed a ‘traditional or normal’ speed of movement (1–2 s for eccentric and 1 s for concentric) or did not control for tempo of movement.

### Participants and Training Status

Four out of the 22 studies recruited trained or ‘technically proficient’ participants. One study defined trained as a minimum of 1 year resistance training [53], whereas another study did not provide a definition [35]. One study required a minimum 1RM in the bench press of 100 kg; however, due to recruitment issues, this was reduced to 60 kg [44]. The fourth study required the participants to be technically proficient in the back squat [45]. One study reported that the participants had previous strength training at recreational level, but underwent no strength training for 3 months leading up to the study [40], and one study accepted participants who were training less than twice per week for 6 months leading up to the study [49].

The remaining studies recruited non-resistance trained participants ranging from 3 months to 5 years without any form of resistance training. 10 studies used University or College students; seven described their participants as ‘healthy’ and four described them as ‘untrained’. The remaining two studies recruited either University or team-sports athletes. The control group across all studies were reported to have ‘maintained normal daily activities’ or to have ‘undertaken no resistance or endurance training’ throughout the duration of the intervention period; however, no study reported how this was controlled for.

### Methodological Quality

The mean ± SD methodological quality rating score was 17.7 ± 2.3 out of a possible 29, with a range of 14–23 (Table 5). Only four studies achieved a methodological quality rating of good, which was categorised as a score of 20 or above [39, 48, 53, 54]. Other studies scored a ‘moderate’ rating. All studies scored 0 for attempting to blind participants from the intervention and its outcomes. It was not possible to determine whether participants were recruited over the same time period and whether the intervention was concealed from participants and administrators across all studies. All studies reported the aims and/or hypotheses; the main outcome measures; the intervention employed; the point estimates of random variability; and employed appropriate statistical analysis. Four studies did not report full participant characteristics [33, 35, 36, 47] and four different studies failed to clearly describe their main findings [37, 39, 47, 49]. It was not possible to determine whether the sample represented the population in one study [34]; however, all studies did recruit both experimental and control groups from the same population. No retrospective unplanned subgroup analyses were reported in any of the studies. Six studies reported adherence or compliance to the intervention [43, 44, 47–49, 53], which was ≥ 92%, whilst 11 studies incorporated supervised training sessions into their interventions.

**Table 5:** Methodological quality evaluation using the modified Downs and Black quality assessment tool

Study	Reporting										External validity			Internal Validity																Total
														Bias							Confounding									
	1	2	3	4	5	6	7	8	9	10	11	12	13	14	15	16	17	18	19	20	21	22	23	24	25	26	27	28	29	
Weiss et al. (1988) [33]	1	1	0	1	1	1	1	0	1	1	1	1	1	0	0	1	1	1	0°	1	1	0°	1	0°	0°	1	1	0	0°	19
Braith et al. (1993) [34]	1	1	1	1	1	1	1	0	0	0	0°	0°	0°	0	0	1	1	1	0°	1	1	0°	1	0°	0°	0°	1	0	0°	14
Moss et al. (1997) [35]	1	1	0	1	1	1	1	0	1	0	1	1	0°	0	0	1	1	1	0°	0	1	0°	0°	0°	0°	1	1	0	1	16
Bell et al. (2000) [36]	1	1	0	1	1	1	1	0	0	0	1	1	0°	0	0	1	1	1	0°	1	1	0°	1	0°	0°	0	1	0	0°	15
Campos et al. (2002) [37]	1	1	1	1	1	0	1	1	1	0	1	1	0°	0	0	1	1	1	0°	1	1	0°	1	0°	0°	1	0	0	0°	17
McBride et al. (2003) [38]	1	1	1	1	1	1	1	0	0	0	1	1	0°	0	0	1	1	1	0°	1	1	0°	0°	0°	0°	0	1	0	0	15
Willoughby (2004) [39]	1	1	1	1	1	0	1	0	1	1	1	1	0°	0	0	1	1	1	1	1	1	0°	1	0°	0°	1	1	0°	1	20
Tricoli et al. (2005) [40]	1	1	1	1	1	1	1	0	1	0	1	1	0°	0	0	1	1	1	1	1	1	0°	1	0°	0°	1	1	0	0°	19
Rana et al. (2008) [41]	1	1	1	1	1	1	1	0	0	1	1	1	0°	0	0	1	1	1	1	1	1	0°	1	0°	0°	0	1	0	1	19
Tanimoto et al. (2008) [42]	1	1	1	1	1	1	1	0	0	0	1	1	0°	0	0	1	1	1	0°	1	1	0°	1	0°	0°	0	1	0	0	16
Terzis et al. (2008) [43]	1	1	1	1	1	1	1	0	1	0	1	1	0°	0	0	1	1	1	0°	1	1	0°	0	0°	0°	0	0	1	0	16
Hartmann et al. (2009) [44]	1	1	1	1	1	1	1	0	0	0	1	1	0°	0	0	1	1	1	0°	1	1	0°	0	0°	0°	0	1	1	1	17
Cormie et al. (2010) [45]	1	1	1	1	1	1	1	0	0	0	1	1	0°	0	0	1	1	1	0°	1	1	0°	1	0°	0°	0	1	0	1	17
Chtourou et al. (2012) [46]	1	1	1	1	1	1	1	0	0	1	1	1	0°	0	0	1	1	1	0°	1	1	0°	1	0°	0°	0	0	0	1	17
Weier et al. (2012) [47]	1	1	0	1	1	0	1	0	0	1	1	1	1	0	0	1	1	1	0°	1	1	0°	1	0°	0°	0	0	1	1	17
Naclerio et al. (2013) [48]	1	1	1	1	1	1	1	0	1	1	1	1	1	0	0	1	1	1	1	1	1	0°	1	0°	1	1	0	1	0°	22
Aguiar et al. (2015) [49]	1	1	1	1	1	0	1	0	1	0	1	1	0°	0	0	1	1	1	0°	1	1	0°	1	0°	0°	1	1	1	1	19
Akagi et al. (2016) [50]	1	1	1	1	1	1	1	0	1	1	1	1	0°	0	0	1	1	1	0°	1	1	0°	1	0°	0°	0	1	0	0°	18
Botton et al. (2016) [51]	1	1	1	1	1	1	1	0	0	0	1	1	0°	0	0	1	1	1	0°	1	1	0°	1	0°	0°	0	1	0	1	17
Wirth et al. (2016) [52]	1	1	1	1	1	1	1	0	0	0	1	1	0°	0	0	1	1	1	0°	1	1	0°	0	0°	0°	0	1	0	1	16
Jarvis et al. (2017) [53]	1	1	1	1	1	1	1	0	1	1	1	1	1	0	0	1	1	1	1	1	1	0°	1	0°	0°	1	1	1	1	23
Souza et al. (2018) [54]	1	1	1	1	1	1	1	1	1	1	1	1	0	0	0	1	1	1	0°	1	1	0°	1	0°	0°	1	1	0	0	20

Items 1–10 are related to reporting, items 11–13 are related to external validity, items 14–20 are related to internal validity (bias), items 21–26 are related to internal validity (confounding), item 27 is related to statistical power, item 28 is related to exercise adherence and item 29 is related to exercise supervision

*1* criteria met, *0* criteria not met, *0°* Item was unable to be determined or scored

## Discussion

The aim of this review was to compare the effectiveness of two load prescriptive methods on maximal strength development. Through a robust systematic search strategy and quality assessment, 22 research articles met the inclusion criteria, with 12 employing a % 1RM prescriptive approach, and the remaining 10 utilising the RM method for prescribing load (Tables 1, 4). The aforementioned strategies of load prescription are widely used across S&C practices, with a large number of resistance training intervention studies also utilising these methods. Nevertheless, this is the first review to compare the two methods against one another to inform practitioners as to the most effective method for developing maximal strength.

The main finding of this review was that both % 1RM and RM prescriptive methods were effective in improving maximal strength. Collectively, all training groups across the 22 included studies improved maximal strength following their interventions (31.3 ± 21.9%; 95% CI 33.1–29.5%; *P *< 0.05) in comparison with their non-training control groups (3.4 ± 4.3%; 95% CI 3.9–2.9%). When comparing maximal strength improvements from the two different methods, the RM target training groups collectively improved by 5.7% more than the relative training groups (Table 3).

However, on closer inspection, the greater increases in strength following the RM method of prescription might be attributed to the 73–114% increase in back squat strength following an 8 week intervention in healthy, untrained males with a mean body mass of 77.8 kg observed in one study (Campos et al. [37]). The post-testing absolute 1RM values for one group equated to 246.5 kg, indicating a relative strength ratio of > 3 × body mass. When comparing to the current powerlifting rankings for the back squat, this level of lower body strength would enable these participants to finish approximately 27th in the 2019 world championships if competing in the back squat alone [55]. Therefore, it is likely that this study is skewing the RM data. Furthermore, no standardisation of technique has been provided for the back squat, thus indicating that a full depth squat might not have been implemented given the loads lifted. This information is vital for readers to fully understand the methods employed, and standardisation within and across research studies needs to be agreed upon.

When removing this particular research article, and then reanalysing the RM results, the mean percentage improvement from pre- to post-testing across the 11 studies remaining fell to 24.2 ± 10.81% (95% CI 23.1–25.3%) (Table 4). This is in agreement with Carroll et al. [25] who directly compared relative prescriptive methods against RM targets and found that a relative daily maximum group was more effective in improving vertical jump, RFD and maximal strength in comparison with the RM group (*p *< 0.05, Hedge’s g = 0.69–1.26). Carroll et al. [25] suggested that a potential build-up of residual neuromuscular fatigue from training to failure and reduction in rapid force production in the RM group might explain the lesser improvements. This idea has been presented on an acute level, in which the time course for recovery has been prolonged following a bout of resistance training to muscular failure [56]. A recent review by Davies et al. [28] observed that no statistically significant differences were evident when comparing training to failure vs. non-failure training. Similarly, Sundstrup et al. [57] highlighted that no greater motor unit recruitment was evident when training to failure vs. heavy loading training. Whilst training to failure may not affect improvements in maximal strength, the prolonged recovery time may be a negative contributing factor. Further investigation is required directly comparing these two methods of load prescription to determine the most appropriate approach across multiple athletic populations and training phases.

The present review highlighted important heterogeneity (such as demographics, testing procedures, and training prescriptions) within the included studies, making inferences about the efficacy of these methods challenging and elucidating consensus difficult. Large variation in the participants recruited (age and training status); training prescriptions employed (sets, reps, load, and rest), exercises prescribed, and the tools used to measure maximal strength (various 1RM procedures, etc.) were evident in the literature. Despite agreement with Carroll et al. [25], such disparity in methodological approaches made comparisons across the 22 included studies difficult and we, therefore, recommend that this initial finding be viewed with caution. More research is perhaps required to evaluate these approaches to load prescription.

Training prescriptions that exceeded 6 weeks in duration appeared to improve maximal strength greater than shorter interventions (32.1–32.7% vs. 27.2%); however, the magnitude of these improvements decreased notably when exceeding this duration (Table 4). For example, McBride [38] found that larger improvements in the leg press exercise across the first 6 weeks compared to the second 6 weeks of training, irrespective of volume (1RM improvements 0–6 weeks: 26.6–27.7% across groups; and 1RM improvements 6–12 weeks across groups: 10.7–18.0%), whilst Cormie [45] found much larger improvements in the back squat at mid-test stage compared to post-test (22.7% vs. 4.5%]. Despite progressive training prescriptions being employed, these data suggest that utilising the same training intervention (e.g., exercises, periodisation approach etc. with small progressions in load prescription) for greater than 6 weeks could result in a plateau in maximal strength development, necessitating variation in training stimuli to elicit further improvement [1–5]. It is also possible that the initial 6 weeks of training would facilitate a rapid increase in neuromuscular adaptations, with hypertrophy becoming more dominant once these have run their course [38]. However, given the interaction between volume and hypertrophic responses to training [30, 32], it would be difficult to make these assumptions when the training frequency prescribed in the included articles in this review did not exceed 3 × week.

Improvements in maximal strength appeared to be influenced by exercise mode (Table 4). When comparing multi-joint, compound exercises (e.g., back squat or clean and jerk) with single-joint, isolation exercises (e.g., seated plantar-flexion or knee extension) greater improvement in maximal strength were evident. Multi-joint, compound exercises require greater neuromuscular recruitment, inter-and-intra-muscle coordination and better utilisation of muscle stabilisers and synergists than smaller, single-joint exercises [2, 6]. It is pertinent to note that the transference of single-joint exercises to sport-specific actions such as jumping and sprinting is limited and that these exercises, therefore, have limited application when training for sport performance [2]. Similarly, our findings highlighted that greater relative improvements in maximal strength were observed in lower body vs. upper body exercises (Table 4), perhaps due to the recruitment of larger muscle groups and exposure to greater loads typical of these exercises.

The training prescriptions (exercises, volume, load and rest) employed within the 22 studies included in this review can be found in Table 2. Large variability in approaches for developing maximal strength was evident across both load prescription methods (% 1RM and RM), with ranges of 1–5 + exercises across a mixture of both single- and multi-joints, volumes of 3–28 reps across 1–6 sets, rest periods of 1–5 min, and intensities ranging from 60 to 120% of 1RM or 3–28 RM targets. This heterogeneity highlights a clear disparity in optimal training prescription for developing maximal strength, making the assessment of effective training prescriptions difficult, and perhaps highlights that improvements can be observed with multiple approaches. Researchers should seek to develop a greater consensus on the more appropriate methods for developing maximal strength within different demographics.

Training recommendations are linked to important underpinning physiological adaptations [1–7] and that the manipulation of loads and volumes can elicit different adaptations [2, 4]. This review, however, indicates that there might be poor agreement about the physiological mechanisms underpinning maximal strength training. Adaptations to the neural system, such as the recruitment of additional or higher threshold motor units [34, 35], the recruitment of more fast twitch muscle fibres (type IIx), greater synchronisation of discharge of motor units [38, 44], greater efferent drive [44], increases in corticospinal excitability coinciding with reductions in short-interval intracortical inhibition [47] or enhanced neural coordination [52, 53], have all been suggested to underpin improvements in maximal strength. In contrast, increases in muscle cross-sectional area, the conversion of muscle fibre types from type IIa to type IIx, changes in pennation angle, and the secretion of growth-promoting hormones [37, 43, 45, 48, 51] have also been suggested to explain maximal strength improvements following training. Whilst disparity in explanations might exist in the literature, this does, however, highlight that maximal strength is a complex quality that can be influenced by both neurological and morphological adaptations. Heterogeneity in physiological measurements (EMG, corticospinal excitability, DEXA scanner, BOD POD, muscle biopsies, blood sampling, or force plate data), the training status and abilities of the participants recruited, and the prescriptions of the training interventions, were noted during our analyses. Such variety in assessment methods, samples, and prescriptions might explain this disparity in physiological explanations offered by the studies included in this review. Further research might be needed to understand and isolate the physiologic mechanisms underpinning the prescriptions of maximal strength.

The majority of studies included in this review (18 articles; Table 1) recruited untrained or detrained participants, most of which ranged from 3 months to 5 years without consistent strength training. Despite this heterogeneity, all studies observed increases in maximal strength in their training groups. Those that recruited resistance-trained athletes (Table 1) observed notable increases in strength, ranging from 6.8 to 27.3%; studies using non-trained participants observed improvements ranging from 2.8 to 114.0% (87.3% when omitting [37]) in magnitude. This supports the suggestion that untrained individuals improve strength to a greater extent and at a faster rate than trained individuals [58]. It is important to note, therefore, that data from untrained individuals might not reflect that of trained individuals and that research findings from one group should not be extrapolated to the other. Trained and untrained individuals respond to training stimuli differently, which can vary based upon their training history and current status [2]. It is thought that untrained individuals will benefit from basic resistance training approaches, whereas trained individuals require more sophisticated methods due to a more developed neuromuscular system [2, 4]. Furthermore, there is growing consensus that a baseline of maximal strength underpins a number of important performance parameters and that certain strength levels might be required prior to undertaking more advanced training methods [1, 2, 4]. Therefore, researchers and practitioners should be cognisant of training status when designing training programmes, and ensure that the methods employed match the training status of the athletes that they are prescribing for. Further research should investigate the use of prescriptive methods on trained and elite individuals specifically.

Often, methods used in practice precede empirical underpinning, and S&C practitioners sometimes utilise strategies before research has validated their efficacy [59]. The availability of other prescriptive methods to S&C coaches and practitioners is apparent in practice; however, the research does not necessarily reflect this. Similarly, recent criticisms of current methods of prescription (% 1RM and RM targets) such as the inflexibility and inaccuracies in training prescriptions following rapid increases in strength or the build-up of residual fatigue [60–63] and the development of new technologies have allowed practitioners to utilise other means for load prescription [64–66]. Subjective methods of autoregulation such as Repetitions in Reserve (RIR) or Ratings of Perceived Exertion (RPE) have been suggested as an alternative strategy to prescribe load [17, 67–70]. Likewise, the utilisation of the measurement of barbell velocity is also evident in practice. Given the strong relationship between load and velocity, individuals are profiled and then associated velocities can be used to manipulate the absolute load lifted each session or each working set [71–76]. Despite these two methods being prevalent in practice, the amount of investigation into their efficacy is limited and warrants significant research in the future.

### Quality Assessment

The quality of the studies included in this review, as assessed by the modified Downs and Black checklist [2, 3], had a mean score of 17.68 ± 2.28, suggesting a moderate rating of methodological quality (Table 5). Four out of the 22 studies were classed as having a good methodology (≥ 20) [39, 48, 53, 54], with the remaining studies being classified as moderate (10–19). Although no studies were methodologically poor, there were still some noteworthy findings. Of the 29 point checklist, only 9 of the criteria were met by all of the studies, with eight of the criteria met by ≤ 5 studies. In accordance with Davies et al. [28], no study reported any adverse effects as a result of the programme intervention prescribed. When researching an intervention, any adverse effects or confounding variables should be reported [30]. This lack of transparency could conceal important biases that affect the quality of this data.

A number of the internal validity criteria were not met by any study. These were: attempting to blind participants, and attempting to blind those measuring the main outcome variables from the intervention. Although, in some cases, this might have improved the quality of the research, blinding participants from a training-programme intervention are difficult, and this might not have affected the overall methodological quality of the evidence [28, 29]. Such issues need to be considered by researchers who use similar checklists when evaluating intervention studies such as these, as the methodological limitations of these tools might lead to erroneous conclusions being drawn about the evidence. Two other criteria not explicitly met or reported by any of the studies were whether participants across multiple intervention or control groups were recruited over the same time period, and whether assignment of groups were concealed from participants and staff until after the intervention was complete. Failure to meet both of these criteria may have increased the risk of selection bias or participants not being placed in appropriate groups [28], and is an important risk of bias in the evidence. This could increase the possibility that a population was sampled until the desired conclusion was reached [28–30].

Only 11 studies reported that the interventions were supervised, and only seven studies reported any exercise adherence data. This is important, as poor adherence could have affected the successful completion of the interventions and impacted the data reported. Full supervision of a training intervention is, therefore, necessary for health and safety purposes, but to also ensure that data are accurate. Indeed, adherence should be recorded to ensure that outliers or suspect results are not due to partial completion and alterations in training frequency between groups [31]. Despite the aforementioned concerns, it should be noted that quality assessment tools that can evaluate strength training interventions are scarce. With a large bias towards clinical trials, a lot of the tools available (Cochrane, PEDro, Downs and Black) do not suit intervention studies in which blinding may be difficult, for example. Therefore, if researchers are to reliably assess methodological quality in the future, a more appropriate and robust tool might be needed if accurate assessments of the evidence are to be made using quality-assessment metrics in applied research such as this.

### Strength and Limitations

The strengths of this review include the systematic nature of the search strategy, which rigorously followed the PRISMA guidelines [26]. The data extraction process and the quality assessment tools employed were all in accordance with previous literature and guidance [26, 28–31]. Despite stringent inclusion criteria, the search terms were inclusive, evidenced by the number of original articles returned (Fig. 1). This inclusive search strategy was purposeful, to draw out as much evidence as possible. However, due to this, the ability to control for things such as programme design, participant characterisation, training status (etc.) became challenging, and might explain the heterogeneous sample, making direct comparisons between some studies challenging. However, this is perhaps also reflective of the wide range of programming tools and methods employed within research (and practice). The heterogeneity of the studies included in this review also prevented any form of meta-analysis to be undertaken, reducing the statistical impact of the findings.

Volume was not controlled for within this review. Previous research has demonstrated a strong dose–response relationship for physical adaptations such as maximal strength [3, 10]. It is possible that without establishing inclusion criteria that controlled for training volume, the application of data presented in this review could be limited. However, the aim of this review was to evaluate methods to prescribe load specifically and that the inclusion criteria of this review were developed to be sensitive to a breadth of literature.

Some studies failed to report all or relevant strength data [e.g., 37, 39, 47], whilst two studies only reported relative (1RM/BM) values [39, 45]. Requests were sent to all authors to provide additional data, with only one providing the necessary information. In some cases, a graph digitizer was, therefore, required to extract the data, potentially reducing the accuracy of some of the values presented in Table 3. Despite this potential limitation, this approach highlights the robust and meticulous methods employed to extract and analyse relevant data.

The need for efficacy trials to include a non-training control group is important to ensure full confidence in the intervention under investigation. This inclusion criterion could have potentially limited the return of some related articles. However, Bishop [77] argues that all efficacy trials (intervention studies) should be characterised by strong control, with a tightly delivered, standardised intervention to a specific, narrowly defined and motivated homogenous group. Indeed, it is this strict control that allows for any effects to be attributed to the intervention under investigation [77]. With this, we did not want to compromise quality for quantity; therefore, the decision to be stringent on the control group was upheld. This further highlights the need for researchers to make every attempt to control their studies as robustly as possible to further develop the quality of research in this area.

### Practical Recommendations

Practitioners should be confident in employing either % 1RM or RM targets as a method of load prescription to improve maximal strength. The two methods, however, have different nuances in strategy and, therefore, are not interchangeable. S&C coaches may favour the % 1RM method, given the greater improvement in maximal strength over the course of progressive intervention (> 4 weeks) evident from this review. If practitioners would prefer a more auto-regulatory method of load prescription, RM targets may be appropriate; however, careful fatigue management would be necessary due to the element of training to failure within this method [22, 56, 57]. In fact, potentially prescribing via % 1RM can allow coaches to better manage the build-up of residual fatigue and prevent a state of unplanned over-reaching. Moreover, practitioners must ensure that the training interventions they prescribe are appropriate for the individuals they work with, utilising quality research as a frame of reference.

The assumption that the % 1RM target method elicits greater strength gains based on the results of this review should also be taken with caution. Whilst a recent study [25] showed that relative prescriptions was more effective at improving jump performance, RFD and maximal strength than RM targets, more research is required in this area, particularly directly comparing these two methods against one another. Practitioners should evaluate the necessity of training to failure and assess the intervention, and subsequently the method of load prescription, on a case-by-case basis dependent on age, training status, periodised approach, and time of season [1, 2, 25].

Despite the effectiveness of the two aforementioned methods, practitioners should still be aware of the potential logistical and physiological flaws when using this method. To administer comprehensive and safe, 1RM assessments with trained or untrained individuals can be difficult due to the proficiency needed in training at high loads, as well as the challenges logistically when employing it with a team of athletes [19, 60]. Practitioners should also take into account the daily fluctuations in force output, strength levels, and residual fatigue that may affect an individual’s daily maximal intensity capabilities [61, 62]. Therefore, considering alternative or additional methods such as velocity or RIR may help maximise load prescription and maximal strength adaptations.

### Future Research

Future research should seek to investigate a direct comparison between % 1RM and RM targets to determine the most effective method of load prescription. Despite being used widely within practice and utilised in isolation across S&C research, the efficacy of these methods has not been investigated and thus requires further attention to evaluate their ability to improve maximal strength. Future research should also examine other common methods of load prescription such as velocity or RIR to provide practitioners with the most effective strategy to improve maximal strength. Researchers should seek to develop research informed guidelines based around training variables related to the development of maximal strength. Guidance on definitions of what constitutes a trained individual is imperative to further the application of research to practice. Importantly, researchers should employ more robust methodologies when investigating the efficacy of training interventions. Furthermore, if methodological quality is to be assessed within the field of S&C, the development of a more appropriate and specific measurement tool may be necessary to ensure valid judgements can be made. Based on the research returned from this review, and the methodological quality assessment we employed, the following guidelines should be followed wherever possible:

Research design recommendations:

- Ensure the testing methods are appropriate for your hypothesis (e.g., if investigating maximal strength, employ a practical and reliable strength assessment).
- Always try to employ a non-training control.
- All groups must be matched in terms of n.
- Any resistance training intervention must be progressive in terms of load, volume, and complexity.
- Resistance training interventions must be clearly described and easy to replicate.
- Data must be clearly displayed with absolute and relative values easily extractable.
- Where possible, create as ‘real world’ a training and testing environment as possible whilst not compromising levels of control.
- Standardise and report testing procedures in full (protocols, movement technique, equipment etc.)
- Recruit participants from the same population across the same time points for multiple experimental or control groups.
- Report exercise adherence and intervention supervision.

## Conclusions

This systematic review demonstrates that prescribing load via a combination of a direct measurement of strength (1RM), and then, submaximal prescriptions is effective in eliciting maximal strength adaptations. Furthermore, the two approaches highlighted in this review, RM targets and relative submaximal percentages (% 1RM), both have a positive impact on maximal strength development in comparison with non-training controls. % 1RM elicited greater improvements in maximal strength (> 4.6%) in comparison with RM targets. More research, however, is needed to fully investigate the efficacy of both these methods, specifically direct comparisons between the two methods. Multi-joint, lower body, compound exercises appear to be more effective in improving maximal strength than their counter-parts. The law of diminishing returns highlights that the magnitude of change in maximal strength decreases following 6 weeks of training. The heterogeneity of the research in this area is evident from this review, and therefore, guidelines are required to help practitioners make informed decisions on the best way to prescribe and programme for their athletes. It is, however, important that practitioners look to utilise the research available to them to ensure appropriate prescriptions can be made, considering such things as training status, age, background etc.

## Electronic supplementary material

Below is the link to the electronic supplementary material.

Supplementary material 1 (DOCX 19 kb)
