# Correction to: The Effectiveness of Two Methods of Prescribing Load on Maximal Strength Development: A Systematic Review

**DOI:** 10.1007/s40279-019-01253-z

**Published:** 2020-01-02

**Authors:** Steve W. Thompson, David Rogerson, Alan Ruddock, Andrew Barnes

**Affiliations:** Academy for Sport and Physical Activity, Sheffield Hallam University, Sheffield, UK

## Correction to: Sports Medicine 10.1007/s40279-019-01241-3

While typesetting the entries of the Table 1 were incorrectly aligned. The correct Table 1 has been copied below.

**Table 1:** Study characteristics

Study	Participants (*n*)	Groups (+ participant numbers (*n*))	Sex (*n*)	Age (years ± SD)	Body mass (kg ± SD)	Stature (cm ± SD)	Resistance training experience	Participant characterisation
Weiss et al. (1988) [33]	56	RT (28)	M (28)	20.8 ± 1.8	NR	NR	NRT < 3 months	Healthy
		C (28)	F (28)					
Braith et al. (1993) [34]	58	RT (47)	M (33)	24.0 ± 4.0	70.1 ± 9.0	174.0 ± 6.3	NRT < 1 year	Untrained
		C (11)	F (25)	25.0 ± 5.0	74.3 ± 14.5	172.6 ± 6.6		
Moss et al. (1997) [35]	31	RT—G90 (9)	M	22.7 ± 3.4	75.8 ± 5.6	179.0 ± 6.8	Well-trained	University students (non-dominant arm = control group)
		RT—G35 (11)		24.0 ± 3.4	83.2 ± 8.8	185.7 ± 8.5		
		RT—G15 (10)		22.9 ± 2.8	78.1 ± 10.4	182.6 ± 6.7		
Bell et al. (2000) [36]	21	RT (11)	M (12)	22.3 ± 3.3	73.4 ± 11.6	176.0 ± 9.3	NRT	University students
		C (10)	F (9)					
Campos et al. (2002) [37]	31	RT—LR (9)	M	21.1 ± 1.5	80.1 ± 8.4	179.8 ± 6.5	NRT < 6 months	Healthy
		RT—IR (7)		20.7 ± 2.9	79.5 ± 7.8	179.6 ± 7.4		
		RT—HR (7)		20.4 ± 3.5	70.2 ± 9.5	174.3 ± 8.6		
		C (5)		31.6 ± 9.8	80.8 ± 23.3	178.1 ± 5.5		
McBride et al. (2003) [38]	28	RT—S1 (9)	F (13) M (15)	22.1 ± 3.4	83.7 ± 29.4	172.8 ± 10.5	NRT (< 6 months)	Untrained
		RT—M6 (9)		20.0 ± 1.22	70.7 ± 23.0	169.4 ± 11.8		
		C (10)		22.4 ± 1.89	70.6 ± 7.8	171.3 ± 7.2		
Willoughby (2004) [39]	22	RT (12)	M	20.9 ± 2.76	78.7 ± 6.2	176.5 ± 7.1	NRT < 6 months	Untrained
		C (10)						
Tricoli et al. (2005) [40]	14	RT (7)	M	22.0 ± 1.5	73.4 ± 10.4	179.4 ± 8.8	NRT < 3 months (trained prior)	College students
		C (7)						
Rana et al. (2008) [41]	16	RT (9)	F	20.6 ± 1.9	64.1 ± 7.9	165.6 ± 4.9	NRT	Untrained
		C (7)		22.9 ± 2.4	72.5 ± 15.0	163.6 ± 4.5		
Tanimoto et al. (2008) [42]	24	RT (12)	M	19.5 ± 0.5	63.8 ± 4.0	174.8 ± 4.3	NRT	Healthy
		C (12)		19.8 ± 0.7	64.2 ± 4.0	174.3 ± 7.2		
Terzis et al. (2008) [43]	17	RT (11)	M	22.0 ± 1.0	85.0 ± 4.0	184.0 ± 3.0	NRT < 1 year	P.E students
		C (6)						
Hartmann et al. (2009) [44]	40	RT—SPP (13)	M	24.31 ± 3.2	84.7 ± 11.2	183.9 ± 7.2	RT in BP (minimum 1RM of 100 kg)	Sport science students
		RT—UP (14)		25.14 ± 4.0	79.4 ± 10.4	177.6 ± 7.5		
		C (13)		24.77 ± 3.1	74.4 ± 12.1	180.5 ± 8.1		
Cormie et al. (2010) [45]	16	RT (8)	M	23.9 ± 4.8	79.8 ± 12.0	180.0 ± 6.4	NRT (Technically proficient in BS)	Healthy
		C (8)						
Chtourou et al. (2012) [46]	30	RT—MTG (10)	M	22.9 ± 1.3	72.0 ± 8.8	180.0 ± 5.0	NRT < 6 months	P.E students
		RT—ETG (10)						
		C (10)						
Weier et al. (2012) [47]	12	RT (6)	M (6) F (6)	20 ± 0.8	NR	NR	NR	University students
		C (6)		22 ± 0.6				
Naclerio et al. (2013) [48]	32	RT—LV (6)	M (20) F (12)	23.3 ± 1.2	66.4 ± 11.0	169.9 ± 8.4	NRT < 5 years	Team sports athletes Soccer (20) (M) Volleyball (12) (F)
		RT—MV (6)		23.3 ± 1.4	71.4 ± 8.5	173.3 ± 7.6		
		RT—HV (8)		23.9 ± 2.0	69.4 ± 12.5	173.0 ± 9.8		
		C (7)		22.1 ± 1.1	71.1 ± 14.2	169.7 ± 6.9		
Aguiar et al. (2015) [49]	18	RT (9)	M	20.9 ± 2.0	73.7 ± 9.4	173.8 ± 6.9	NRT < 6 months	Healthy
		C (9)		20.0 ± 1.8	75.0 ± 8.8	176.4 ± 8.1		
Akagi et al. (2016) [50]	23	RT (13) C (10)	M	22.1 ± 1.1	61.4 ± 5.8	170.6 ± 5.8	NRT upper body (< 6 months)	Healthy
Botton et al. (2016) [51]	43	RT—UG (14)	F	24.8 ± 1.4	60.8 ± 6.4	163.0 ± 6.5	NRT < 3 months	Healthy
		RT—BG (15)		24.3 ± 3.7	57.0 ± 4.8	160.2 ± 5.8		
		C (14)		22.7 ± 2.8	58.0 ± 5.7	163.6 ± 6.2		
Wirth et al. (2016) [52]	120	RT—SQ (43)	M	23.7 ± 2.7	81.6 ± 9.8	181.7 ± 7.5	NR	Students
		RT—LP (40)		23.8 ± 2.3	80.5 ± 8.1	180.1 ± 7.0		
		C (37)		25.1 ± 2.1	78.2 ± 8.5	181.0 ± 5.7		
Jarvis et al. (2017) [53]	21	RT (11) C (10)	M (15) F (6)	27.5 ± 3.2	72.7 ± 18.0	169.6 ± 10.3	RT > 1 year	Collegiate athletes
				27.2 ± 3.4	76.4 ± 11.5	176.2 ± 7.9		
Souza et al. (2018) [54]	33	RT—NP (8)	M	25.6 ± 6.3	79.5 ± 13.0	172.8 ± 6.1	NRT (< 6 months)	College students
		RT—TP (9)		25.0 ± 7.0	76.0 ± 9.9	175.3 ± 5.7		
		RT—UP (8)		24.4 ± 5.2	74.9 ± 4.2	176.8 ± 5.3		
		C (8)		25.1 ± 3.3	76.8 ± 11.7	173.6 ± 6.8		

*Mean ± SD* standard deviation

*1RM* 1 repetition maximum, *BG* bilateral training group, *C* control, *cm* centimetres, *BP* bench press, *BS* back squat, *ETG* evening training group, *F* female, *G15* 15% load group, *G35* 35% load group, *G90* 90% load group, *HR* high-repetition group, *HV* high volume, *IR* intermediate-repetition group, *kg* kilograms, *LP* leg press group, *LR* low-repetition group, *LV* low volume, *M* male, *M6* six set training group, *MTG* morning training group, *MV* moderate volume, *n* number, *NP* non-periodised group, *NRT* no resistance training, *NR* not reported, *P.E* Physical Education, *RT* resistance training, *S1* 1 set training group, *SPP* strength-power periodisation, *SQ* squat group, *TP* traditional periodisation group, *UG* unilateral training group, *UP* daily undulating periodised group

The original article has been corrected.

