# Supplementary material for: The Effectiveness of Two Methods of Prescribing Load on Maximal Strength Development: A Systematic Review
Source: Sports Med. 2019 Dec 11;50(5):919–38. doi: 10.1007/s40279-019-01241-3 (PMC7142036; doi:10.1007/s40279-019-01241-3)
Supplement: Supplementary file 1 — Supplementary material 1 (DOCX 19 kb) [file 40279_2019_1241_MOESM1_ESM.docx]

The effectiveness of three common methods of prescribing intensity on maximal strength development: a systematic review.

Steve W. Thompson^1^, David Rogerson^1^, Alan Ruddock^1^, Andrew Barnes^1^

^1^Academy for Sport and Physical Activity, Sheffield Hallam University, Sheffield, United Kingdom

Corresponding Author:

Steve W. Thompson

[s.w.thompson@shu.ac.uk](mailto:s.w.thompson@shu.ac.uk)

+447801997099

ORCID I.D:

SW Thompson - 0000-0001-7674-3685

D Rogerson - 0000-0002-4799-9865

A Ruddock – 0000-0002-7001-9845

A Barnes - 0000-0001-8262-5132

**Electronic Supplementary material**

Modified Downs and Black methodological assessment checklist [31]

| **Reporting** | **Score** |
| --- | --- |
| 1. Is the hypothesis/aim/objective of the study clearly described? | 0 – 1 |
| 1. Are the main outcomes to be measured clearly described in the Introduction or Methods section? | 0 – 1 |
| 1. Are the characteristics of the participants included in the study clearly described? | 0 – 1 |
| 1. Are the interventions of interest clearly described? | 0 – 1 |
| 1. Are the distributions of principal confounders in each group of participants to be compared clearly described? | 0 – 1 |
| 1. Are the main findings of the study clearly described? | 0 – 1 |
| 1. Does the study provide estimates of the random variability in the data for the main outcomes? | 0 – 1 |
| 1. Have all important adverse events that may be a consequence of the intervention been reported? | 0 – 1 |
| 1. Have the characteristics of participants lost to follow-up been described? | 0 – 1 |
| 1. Have actual probability values been reported (e.g. 0.035 rather than <0.05) for the main outcomes except where the probability value is less than 0.001? | 0 – 1 |
| **External validity** |  |
| 1. Were the subjects asked to participate in the study representative of the entire population from which they were recruited? | 0 – 1 |
| 1. Were those subjects who were prepared to participate representative of the entire population from which they were recruited? | 0 – 1 |
| 1. Were the staff, places, and facilities where the participants were treated, representative of the treatment the majority of participants receive? | 0 – 1 |
| **Internal validity - bias** |  |
| 1. Was an attempt made to blind study participants to the intervention they have received? | 0 – 1 |
| 1. Was an attempt made to blind those measuring the main outcomes of the intervention? | 0 – 1 |
| 1. If any of the results of the study were based on “data dredging”, was this made clear? | 0 – 1 |
| 1. In trials and cohort studies, do the analyses adjust for different lengths of follow-up of participants, or in case-control studies, is the time period between the intervention and outcome the same for cases and controls? | 0 – 1 |
| 1. Were the statistical tests used to assess the main outcomes appropriate? | 0 – 1 |
| 1. Was compliance with the intervention/s reliable? | 0 – 1 |
| 1. Were the main outcome measures used accurate (valid and reliable)? | 0 – 1 |
| **Internal validity – confounding (selection bias)** |  |
| 1. Were the participants in different intervention groups (trials and cohort studies) or were the cases and controls (case-control studies) recruited from the same population? | 0 – 1 |
| 1. Were study participants in different intervention groups (trials and cohort studies) or were the cases and controls (case-control studies) recruited over the same period of time? | 0 – 1 |
| 1. Were study participants randomised to intervention groups? | 0 – 1 |
| 1. Was the randomised intervention assignment concealed from both participants and health care staff until recruitment was complete and irrevocable? | 0 – 1 |
| 1. Was there adequate adjustment for confounding in the analyses from which the main findings were drawn? | 0 – 1 |
| 1. Were losses of participants to follow-up taken into account? | 0 – 1 |
| 1. Did the study have sufficient power to detect a clinically important effect where the probability value for a difference being due to change is less than 5%? 2. Were exercise sessions supervised? | 0 – 1  0 – 1 |
| 1. Was exercise adhered to? | 0 – 1 |
